# Clustering of malaria in households in the Greater Mekong Subregion: operational implications for reactive case detection

**DOI:** 10.1186/s12936-021-03879-9

**Published:** 2021-08-26

**Authors:** Mavuto Mukaka, Pimnara Peerawaranun, Daniel M. Parker, Ladda Kajeechiwa, Francois H. Nosten, Thuy-Nhien Nguyen, Tran Tinh Hien, Rupam Tripura, Thomas J. Peto, Koukeo Phommasone, Mayfong Mayxay, Paul N. Newton, Mallika Imwong, Nicholas P. J. Day, Arjen M. Dondorp, Nicholas J. White, Lorenz von Seidlein

**Affiliations:** 1 Mahidol Oxford Tropical Medicine Research Unit, Faculty of Tropical Medicine, Mahidol University, Bangkok, Thailand; 2 Mahidol Oxford Tropical Medicine Research Unit, Faculty of Tropical Medicine, Mahidol University, Bangkok, Thailand; 3 Centre for Tropical Medicine and Global Health, Nuffield Department of Medicine, University of Oxford, Oxford, UK; 4 Department of Population Health and Disease Prevention, University of California, Irvine, USA; 5 Shoklo Malaria Research Unit, Mahidol-Oxford Tropical Medicine Research Unit, Faculty of Tropical Medicine, Mahidol University, Mae Sot, Thailand; 6 Oxford University Clinical Research Unit, Wellcome Trust Major Oversea Programme, Ho Chi Minh City, Vietnam; 7 Department of Global Health, Amsterdam University Medical Centers, Amsterdam, Netherlands; 8 Lao-Oxford-Mahosot Hospital-Wellcome Trust Research Unit (LOMWRU), Microbiology Laboratory, Mahosot Hospital, Vientiane, Lao PDR; 9 Amsterdam Institute for Global Health & Development, Amsterdam, Netherlands; 10 Institute of Research and Education Development, University of Health Sciences, Vientiane, Lao PDR; 11 Department of Molecular Tropical Medicine and Genetics, Faculty of Tropical Medicine, Mahidol University, Bangkok, Thailand

## Abstract

**Background:**

Malaria reactive case detection is the testing and, if positive, treatment of close contacts of index cases. It is included in national malaria control programmes of countries in the Greater Mekong Subregion to accelerate malaria elimination. Yet the value of reactive case detection in the control and elimination of malaria remains controversial because of the low yield, limited evidence for impact, and high demands on resources.

**Methods:**

Data from the epidemiological assessments of large mass drug administration (MDA) studies in Myanmar, Vietnam, Cambodia and Laos were analysed to explore malaria infection clustering in households. The proportion of malaria positive cases among contacts screened in a hypothetical reactive case detection programme was then determined. The parasite density thresholds for rapid diagnostic test (RDT) detection was assumed to be > 50/µL (50,000/mL), for dried-blood-spot (DBS) based PCR > 5/µL (5000/mL), and for ultrasensitive PCR (uPCR) with a validated limit of detection at 0.0022/µL (22/mL).

**Results:**

At baseline, before MDA, 1223 *Plasmodium* infections were detected by uPCR in 693 households. There was clustering of *Plasmodium* infections. In 637 households with asymptomatic infections 44% (278/637) had more than one member with *Plasmodium* infections. In the 132 households with symptomatic infections, 65% (86/132) had more than one member with *Plasmodium* infections. At baseline 4% of households had more than one *Plasmodium falciparum* infection, but three months after MDA no household had more than one *P. falciparum* infected member. Reactive case detection using DBS PCR would have detected ten additional cases in six households, and an RDT screen would have detected five additional cases in three households among the 169 households with at least one RDT positive case. This translates to 19 and 9 additional cases identified per 1000 people screened, respectively. Overall, assuming all febrile RDT positive patients would seek treatment and provoke reactive case detection using RDTs, then 1047 of 1052 (99.5%) *Plasmodium* infections in these communities would have remained undetected.

**Conclusion:**

Reactive case detection in the Greater Mekong subregion is predicted to have a negligible impact on the malaria burden, but it has substantial costs in terms of human and financial resources.

**Supplementary Information:**

The online version contains supplementary material available at 10.1186/s12936-021-03879-9.

## Background

Clustering of malaria infections in compounds and households has been reported in most endemic regions [1–11]. Several malaria control and elimination strategies focus on household clustering which, for the purposes of this study, is defined as households where more than one member was found to have a patent or sub-patent *Plasmodium* infection. The principle approach is reactive case detection. After index cases of malaria are identified, their household members, neighbours, and other contacts are screened by RDT or microscopy and treated with anti-malarials if they test positive [12]. This approach was supported in sub-Saharan Africa by a recent analysis of Demographic and Health Surveys (DHS) data which found strong evidence of household clustering of *Plasmodium falciparum* infections in children [13]. As malaria control succeeds, and the prevalence of *Plasmodium falciparum* decreases, the remaining infections tend to cluster in households [13]. A recent study from Namibia found that indoor residual insecticide spraying and presumptive anti-malarial treatment of household members of a *P. falciparum* malaria index case significantly reduced malaria transmission [14]. This is at least in part because in sub-Saharan Africa the endophilic–endophagic and mainly nocturnal *Anopheles* species tend to stay within human habitations after a blood meal and infect other household and community members during subsequent blood meals. The situation is substantially different in regions with predominantly exophilic–exophagic *Anopheles* species, such as South East Asia where outdoor biting immediately after dusk and early morning hours is responsible for a large fraction of malaria transmission. Household members, co-workers and co-travellers may become infected as a result of shared daytime and occupational exposures, but living in the same house as a malaria patient may not be as important a risk factor for becoming infected. Yet targeting of anti-malarial interventions to the households of *P. falciparum* and *Plasmodium vivax* malaria index cases is thought to have played an important role in the successful malaria elimination from China. The Chinese “1-3-7” approach includes reporting of confirmed and suspected malaria cases within 1 day, case confirmation and classification within 3 days and, if local transmission is considered possible, targeted action to detect other infections and to reduce the chance of onward transmission must be completed within 7 days [12]. An adaptation of the “1-3-7” approach, named “case investigation focus investigation and response” (CIFIR) is now being strongly promoted in South-East Asia, although there is only weak evidence in its support, and there are many important differences in malaria epidemiology, accessibility and control programme resources and capability between China and the South-East Asian region.

To assess clustering of *Plasmodium* infections in the Greater Mekong Subregion (GMS) and the potential impact of reactive case detection strategies within the household of the index case, data from surveys conducted in the context of prospective evaluations of mass drug administrations (MDA) were analysed (a) to document clustering in households, using the data from the different MDA rounds, (b) to calculate the potential yield of RACD, using only M0 baseline data, and (c) to evaluate the impact of MDA on clustering.

## Methods

The data for the current study come from the baseline survey and follow-up surveys conducted during large cluster randomized trials in 16 villages in rural Myanmar, Vietnam, Cambodia, and Lao PDR conducted between May 2013 and June 2017 [15, 16]. These villages were selected as being generally representative of the region, and having a relatively high prevalence of symptomatic malaria or *Plasmodium* infections.

### Surveys

Starting immediately before the MDA (M0) and then at 3-month intervals all consenting residents in the study villages aged 6 months and older were followed. The study teams recorded the presence of all household members during the preceding 3 months. Additional information was collected in 2-weekly intervals, including the skin surface or tympanic membrane temperature of all household members. If the skin surface temperature exceeded 38.0 °C or the tympanic membrane temperature exceeded 37.5 °C a rapid diagnostic test for *Plasmodium* infections was performed (Myanmar, Lao PDR, and Vietnam: SD Bioline Malaria Ag P.f/Pan POCT, Standard Diagnostics, Yongin-si, Republic of Korea; Cambodia: Healgen Malaria P. falciparum/Pan 1-step RDT, Zhejiang Orient Biotech, China). If positive, the patient was treated according to national treatment guidelines. Irrespective of signs and symptoms, venous blood samples were collected from all participants; 500 μL from children and 3 mL from those over 5 years of age.

### Trial description

The aim of the MDA trials was to assess the effectiveness, safety, tolerability, and acceptability of mass administrations of three rounds of dihydroartemisinin-piperaquine (DHA-PPQ) with a single low dose primaquine (SLD PQ). Four villages were selected in each country based on their representativeness in terms of regional ecology, population, and human behaviour. During a baseline survey, study teams assigned a unique identification number to each household and member of the household. During subsequent quarterly surveys, the study teams recorded the number of people sleeping in each household during the night before the survey. Overall, 67% of the residents in the study villages participated in at least 3 of the 5 possible surveys, and 32% participated in all 5 surveys. The mass drug administrations (MDAs) were conducted at months 0, 1, 2 in intervention MDA villages. Control villages remained untreated during the study period analysed here. The MDA intervention was allocated by restricted randomization within in each country within two pairs of villages matched for geographical proximity and parasite prevalence. Of the 4423 people residing during M0, M1, and M2 in the 8 intervention villages 3790 (86%) completed at least one round (3 doses) of MDA; 635 (14%) completed only a single round of anti-malarials, 635 (14%) completed 2 rounds, and 2520 (57%) completed all 3 rounds. The 4135 residents in control villages were invited to participate in cross-over MDAs at the end of the study.

### Laboratory investigations

The study team stored blood samples in a cool box in the field and transported the samples within 12 h to the local laboratory. Blood samples from all survey participants were evaluated using standard microscopy and malaria RDTs. Microscopists who had at least 5 years’ experience and/or were confirmed to be Level 2 or higher, as assessed by a standard WHO 55 slide set, performed the standard microscopy. They counted the number of parasites per 500 white blood cells on Giemsa-stained peripheral blood thick films. After separation of EDTA anti-coagulated plasma, buffy coat, and packed red blood cells, samples were frozen and stored at − 80 °C. The study teams transported frozen samples from Myanmar, Cambodia, and Lao PDR monthly on dry ice to the molecular laboratory in Bangkok, Thailand, and the samples from the Vietnam sites to Ho Chi Minh City, Vietnam, for DNA extraction and high-volume ultrasensitive quantitative PCR (uPCR) as described previously [17]. The lower limit of accurate quantitation using this method is 22 parasites/mL of whole blood, and for accuracy and specificity, this was set as the limit of detection. After quantitation of *Plasmodium* genus genome equivalents the *Plasmodium* species in uPCR positive samples was determined using nested PCR specific to *P. falciparum* (microsatellite marker Pk2), *P. vivax* (microsatellite marker 3.502), and *Plasmodium malariae* (18s rRNA) as described previously [18]. Positive samples for which there was insufficient DNA for species identification—or where no amplification was obtained in this second step—were reported as being of indeterminate species (*Plasmodium* spp.).

### Definitions and analytical methods

Individuals registered within a household and sharing the same household number were defined as household members. The individuals who did not have a household number were excluded from the main analysis (Additional file 1: Table S1 compares individuals in households with and without numbers). A sub-patent *Plasmodium* infection was defined as a positive uPCR result in the absence of a positive RDT result i.e. someone with parasitaemia, but who would not be detected by screening with an RDT. A patent malaria case was defined as a positive RDT result (confirmed by uPCR). Thus, *Plasmodium* infections could be asymptomatic and either below or above the detection limit for microscopy or routine RDT, or could be symptomatic (i.e. a malaria episode). As mentioned in the introduction, for the purposes of this study a cluster is defined as members of a household in which more than one member was found to have a patent or sub-patent *Plasmodium* infection. Only households in which there was symptomatic malaria would be investigated in a reactive case detection approach using RDTs. In the analysis index cases were, therefore, defined based on the results of their RDT results and not based on parasite densities which were used for the prediction of yields (below). The proportions of households in which clustering of *Plasmodium* infections occurred were assessed at the time of baseline survey M0. The impact of the MDA on clustering of *Plasmodium* infections was explored using data from the subsequent four surveys.

### Prediction of yields from reactive case detection

Yield of reactive case detection was predicted from the proportion of cases that would have been identified among potentially screened contacts i.e. the number of additional cases that would have been identified by screening household members around an index case using different methods. This was then compared with the total numbers of asymptomatic and symptomatic cases in each village to calculate the likely overall impact of reactive case detection on the total burden of malaria. The lowest parasite density detectable by the RDTs was assumed to be > 50,000/mL (i.e. 50/μL—which is the same level as for microscopy), and for dried-blood-spot (DBS) based PCR was assumed to be > 5000/mL (i.e. 5/μL—or ten times more sensitive than RDTs or microscopy) [19]. Only data collected during the baseline survey, prior to the MDA, were used for the yield analysis because the intervention markedly reduced the prevalence of malaria. The yield of reactive case detection was estimated through the following steps:

- the number of households with a member who had a positive RDT which would have triggered a reactive case detection investigation. It was assumed conservatively that any positive RDT would have become symptomatic whether or not symptoms were present at the time of survey.
- among those households the number of households with one or more *Plasmodium* infected members were counted in households that had more than one member.
- out of those households the number of households with *Plasmodium* infections of sufficient parasite density to be detectable by DBS-PCR (parasite density > 5000/mL) or by RDT (parasite density > 50,000/mL) was estimated.

The yield was then calculated as cases detectable by DBS-PCR and RDT, respectively minus one (the index case) divided by the number household members minus the index cases detected in (b).

The study teams collected survey data on case record forms and entered the data on smartphones before exporting them into OpenClinica (OpenClinica, Waltham, MA, US). Analyses were performed in Stata 15.0 (StataCorp, College Station, Texas, US). A Poisson regression model was used to estimate the uPCR and RDT yields and the yields were expressed as percentages. The 95% confidence intervals were also calculated from this model. There was no need to adjust for clustering as these percentages are already cluster level summaries, that is, counts per household.

## Results

**Fig. 1:**
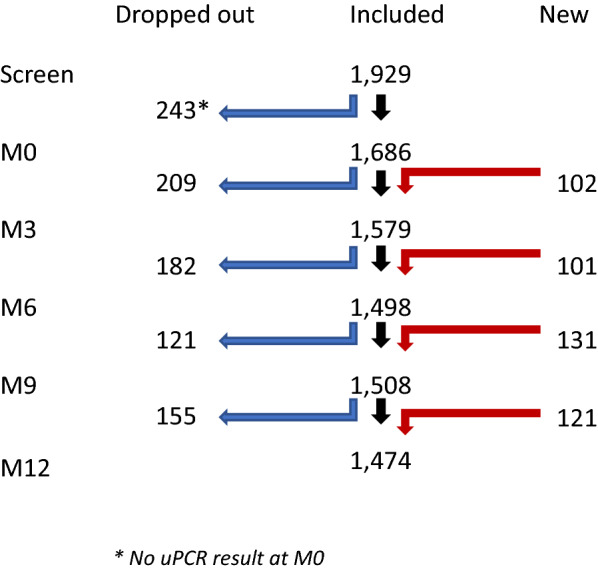
Number of households included, dropped-out and were newly added to the dynamic cohort. The total number of households was 1929 of which 176 households had data only for M0 or had no valid qPCR result. The remaining 1753 households had data available for M3, M6, M9 or M12

At the start of the study (M0, baseline survey) 8445 residents were surveyed. Of these, 6269 (75%) residents had a known household number and a uPCR result. These residents lived in 1686 households which participated in the study. The study cohort was dynamic with households joining and leaving the study (Fig. 1—assembly of study villages). In total 1753 households had data available for M3, M6, M9 or M12. Half of the households, 50% (869/1753) were in villages where MDAs were conducted, and the remainder were in control villages. Out of the 1753 participating households 400 (23%) were in Myanmar, 666 (38%) in Vietnam, 402 (23%) in Cambodia, and the remaining 285 (16%) in Laos (Table 1). During the five quarterly cross-sectional surveys a total of 7745 households were visited. At the baseline survey (M0), 1223/6296 (19%) residents living in 693/1686 (41%) households were found to have *Plasmodium* infections; 171/1223 (14%) residents were symptomatic and the remaining 1052 (86%) were asymptomatic. Of the 1223 infections 289 were identified as *P. falciparum*, 557 as *P. vivax*, 125 as mixed *P. falciparum* and *P. vivax* infections and 252 *Plasmodium* spp*.* (Additional file 1: Table S1A). An additional 19 individuals with *Plasmodium* infections did not have a registered household number and were excluded from the analysis. The species and densities of the *Plasmodium* infections of individuals with and without household number were similar (Additional file 1: Table S1B).

**Table 1:** Characteristics of households at baseline survey M0

Village #	Country	(A) Households	(B) Households with any member with *Plasmodium* infection	(C) Households with > 1 member with *Plasmodium* infection	Prevalence of *Plasmodium* infection in households (B/A) (%)	Probability of having > 1 *Plasmodium* infection in household (C/B)
CM01	Cambodia	90	30	12	33	0.40
CM02	Cambodia	73	15	3	21	0.20
CM03	Cambodia	82	37	11	45	0.30
CM04	Cambodia	123	24	6	20	0.25
LAO01	Laos	69	61	53	88	0.87
LAO02	Laos	76	23	7	30	0.30
LAO03	Laos	78	31	10	40	0.32
LAO04	Laos	59	15	2	25	0.13
MM1	Myanmar	133	85	45	64	0.53
MM2	Myanmar	65	39	17	60	0.44
MM3	Myanmar	81	58	24	72	0.41
MM4	Myanmar	111	82	51	74	0.62
VT1	Vietnam	213	70	15	33	0.21
VT2	Vietnam	90	27	6	30	0.22
VT3	Vietnam	194	46	10	24	0.22
VT4	Vietnam	149	50	16	34	0.32
	Total	1686	693	288	41	0.42

### Evidence of clustering

**Fig. 2:**
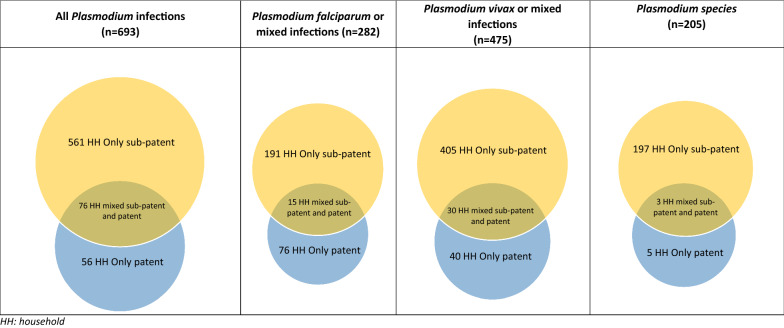
Venn diagrams illustrating households with patent only, sub-patent only or mixed patent and sub-patent, uPCR-confirmed *Plasmodium* infections. *HH* household

**Fig. 3:**
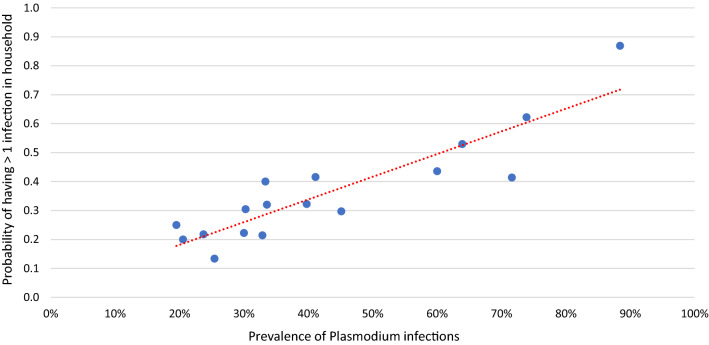
Correlation between parasite prevalence and the probability of having more than one infection in the household in 16 villages surveyed at M0 (R = 0.90) Trendline indicated as red dots

Most (81%; 561/693) of the households with *Plasmodium* infections had only sub-patent infections, the remaining 8% (56/693) and 11% (76/693) had only patent infections or mixed (patent and sub-patent) infections, respectively (Table 2; Fig. 2). In the 637 households with sub-patent infections (mixed or only sub-patent infections) 44% (278/637) had additional members with *Plasmodium* infections. Overall, 12% (202/1686) of all households had two or more members with sub-patent infections only, but only 1% (10/1686) had households with two or more patent infections only. In the 132 households in which the index case was patent 65% (86/132) had additional members with *Plasmodium* infections. The breakdown by *Plasmodium* species is shown in Table 2. There was a strong direct correlation between parasite prevalence in a village and the probability of having more than 1 infection in the household in 16 villages surveyed at M0 (Table 1, Fig. 3; R = 0.90).

**Table 2:** Evidence of clustering of sub-patent and patent, uPCR-confirmed *Plasmodium* infections in households in study villages at baseline surveys M0

All	Households	All	%	%	Pf	%	%	Pv	%	%	P. spp.	%	%
		1686	100%		1686	100%		1686	100%		1686	100%	
1. Households with only sub-patent infections	Households with any infection	561	33	100	191	11	100	405	24	100	197	12	100
	Households with a single infected member	359	21	64	140	8	73	303	18	75	166	10	84
	Households with two or more infected members	202	12	36	51	3	27	102	6	25	31	2	16
2. Households with only patent infections	Households with any infection	56	3	100	76	5	100	40	2	100	5	0	100
	Households with a single infected member	46	3	82	64	4	84	36	2	90	0	0	0
	Households with two or more infected members	10	1	18	12	1	16	4	0	10	5	0	100
3. Households with mixed patent and sub-patent infections	Households with any infection	76	5	100	15	1	100	30	2	100	3	0	100
	Households with a single infected member	0	0	0	0	0	0	0	0	0	0	0	0
	Households with two or more infected members	76	5	100	15	1	100	30	2	100	3	0	100
Total number of households with *Plasmodium* infection	Households with any infection	693	41	100	282	17	100	475	28	100	205	12	100
	Households with a single infected member	405	24	58	204	12	72	339	20	71	171	10	83
	Households with two or more infected members	288	17	42	78	5	28	136	8	29	34	2	17
Total sub-patent infection (including households with both a/symptomatic infections)	Households with any infection	637	38	100	206	12	100	435	26	100	200	12	100
	Households with a single infected member	359	21	56	140	8	68	303	18	70	166	10	83
	Households with two or more infected members	274	16	44	66	4	32	132	8	30	34	2	17
Total patent infection (including households with both a/symptomatic infections)	Households with any infection	132	8	100	91	5	100	70	4	100	8	0	100
	Households with a single infected member	46	3	35	64	4	70	36	2	51	0	0	0
	Households with two or more infected members	86	5	65	27	2	30	34	2	49	8	0	100

*PfP. falciparum* or mixed infection, *Pv* *P. vivax* or mixed infection

### The effect of MDA on clustering

**Fig. 4:**
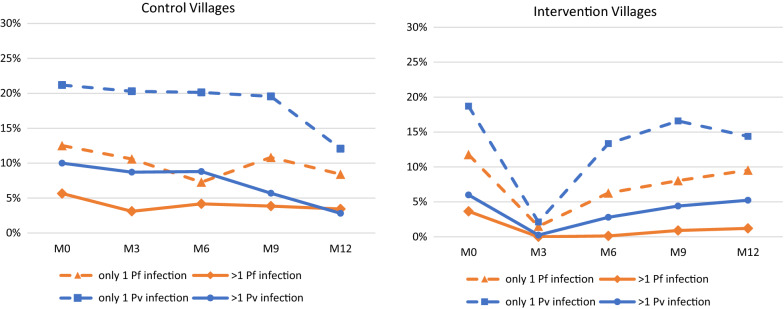
The prevalence of households with multiple *P. falciparum* or mixed infections or *P. vivax* or mixed infections at the time of the cross-sectional survey. Pf means *P. falciparum* or mixed infection; Pv means *P. vivax* or mixed infection

The MDA had a major impact on clusters of *Plasmodium* infections in the intervention villages (Fig. 4). Whereas at baseline 4% of households had more than one *P. falciparum* infection, by M3 no household had multiple *P. falciparum* infections and by M12 only 1% of households had multiple *P. falciparum* infections. The initial effect was similar on households with multiple *P. vivax* infections, which dropped from 6 to 0% at M3, but quickly rebounded and was back to 5% by M12. In the control villages the prevalence of households with multiple *P. falciparum* infections dropped slightly from 6% at M0 to 3% at M12. The corresponding prevalence of households with multiple *P. vivax* infections was 10% at M0 and fell to 3% at M12.

### Potential yield of reactive case detection

**Fig. 5:**
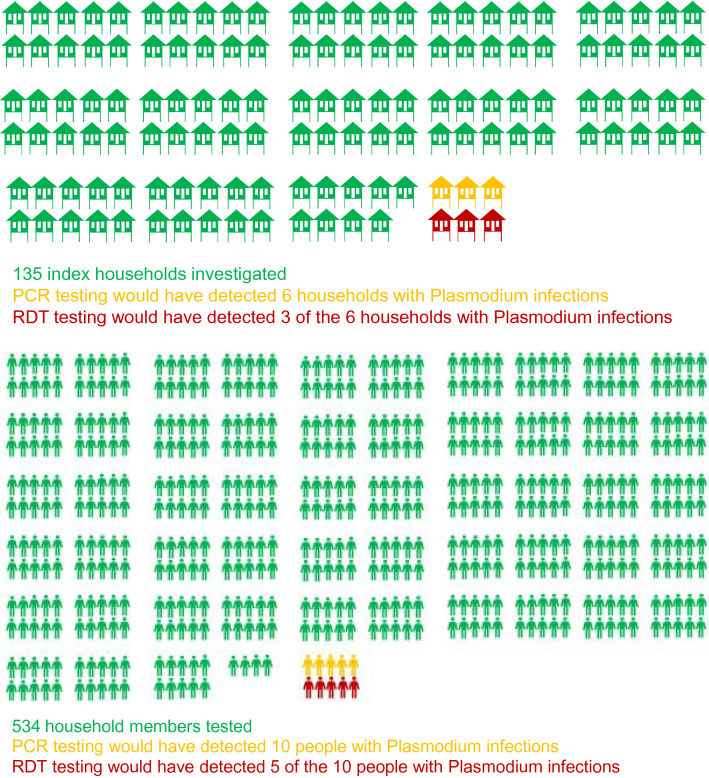
The yield of reactive case detection in the GMS

At the time of the baseline surveys 169 out of 1686 households (10%) had ≥ 1 RDT positive household member (Table 3). Among those 169 households with an index case there were 135 households with more than one member comprising a total of 669 household members that had at least one *Plasmodium* infection (534 members after excluding index cases). Of these 135 households, 6 households had 10 infected residents, which could have been detected by a DBS-PCR with a lower limit of detection 5000/mL and 3 households had 5 infected residents detectable by a RDT with a lower limit of detection of 50,000/mL. Thus, reactive case detection in these households DBS-PCR would have detected 10 additional cases and RDTs would have detected 5 additional cases. The direct yield of reactive case detection using DBS-PCR would have been 1.9% (10/534: 95% CI 0.9 to 3.4) based on 10 cases detected among 534 tested participants (Fig. 5). Using RDT the yield would have been 0.9% (95% CI 0.3 to 2.2) based on the detection of 5 cases among 534 tested, or 107 people (i.e. 534/5) would have to be tested by RDT to detect one additional *Plasmodium* infection. The numbers stratified by *Plasmodium* species are shown in Table 3. Critically, only 5 out of 1052 (0.5%) prevalent *Plasmodium* infections at M0 would have been detected and the remaining 1047/1052 (99.5%) *Plasmodium* infections would have remained undetected using reactive case detection with RDTs as diagnostic test.

**Table 3:** The potential yield of reactive case detection using RDTs in study villages at the time of baseline surveys M0 (see also Fig. 5 illustrating the yield of reactive case detection in the GMS)

	All^a^	Pf	Pv	P. spp.^b^
(a) Households with a RDT positive household member (#members)	169 (773)	124 (564)	71 (368)	NA
(b1) Households with a *Plasmodium* infected member which density reported which had > 1 HH member residing in the HH (#all members)	135 (669)	80 (401)	58 (325)	34 (213)
(b2) Households with a *Plasmodium* infected member which density reported which had > 1 household member residing in the household (#member excluded 1 index case from each household)	135 (534)	80 (321)	58 (267)	34 (179)
2 to 3 household members residing in the household (#all member)	37 (96)	21 (54)	13 (35)	4 (11)
4 to 5 household members residing in the household (#all member)	59 (269)	34 (155)	23 (105)	15 (64)
≥ 6 household members residing in the household (#all member)	39 (304)	25 (192)	22 (185)	15 (138)
(c) Households with a second case PCR detectable *Plasmodium* infection (#non-index asymptomatic cases)	6 (10)	2 (4)	4 (4)	NA
(d) Households with second case RDT detectable *Plasmodium* infection (#non-index asymptomatic cases)	3 (5)	1 (3)	2 (2)	NA
(e) Yield PCR; n/N, % (95% CI)	10/534, 1.9% (0.9 to 3.4)	4/534, 0.7% (0.2 to 1.9%)	4/534, 0.7% (0.2 to 1.9%)	NA
(f) Yield RDT; n/N, % (95% CI)	5/534, 0.9% (0.3 to 2.2)	3/534, 0.6% (0.1 to 1.6%)	2/534, 0.4% (0.05 to 1.6%)	

The yield was estimated through the following steps: (a) the number of households with a member who had a positive RDT which could trigger a reactive case detection investigation. (b) Among those households in (a) the number of households with one or more additional members. (c) Of those households the number of households with Plasmodium infections of sufficient parasite density to be detectable by DBS-PCR (parasite density > 5000/mL) or by RDTs (parasite density > 50,000/mL) was estimated. The yield was then calculated as DBS-PCR detectable cases and RDT respectively minus index cases divided by the number household members minus index cases detected in (b). The number of househfold members are indicated in brackets (n)

*PfP. falciparum* or mixed infection, *Pv* *P. vivax* or mixed infection, *NA* not applicable

^a^Included *Plasmodium* spp.

^b^This column was defined using uPCR only

## Discussion

The study provides evidence of household clustering of *Plasmodium* infections in 16 villages in the malaria endemic areas of four countries in the GMS. Most of these clusters comprised asymptomatic individuals with low parasite density infections (below the limits of detection by microscopy or RDTs, and often below the density detectable by standard dried blood spot PCR). These asymptomatic infections would normally go undetected, but they would likely sustain malaria transmission in the area. Overall, 12% of all households had two or more members with asymptomatic sub-patent infections only. Almost half (45%) of all the asymptomatic *Plasmodium* infections were found in clusters of two or more infected members per household. In contrast, clustering of patent infections was unusual; only 1% had households with two or more patent infections. The mass drug administrations substantially reduced the number of households with multiple *Plasmodium* infections, in particular the clusters of *P. falciparum* infections.

Reactive case detection is being promoted to control and eliminate malaria in the GMS. It faces two major challenges, which make it highly unlikely to have a significant impact on the incidence or prevalence of malaria. The case investigations are labour-intensive and malaria cases tend to occur in remote areas, which are often hard to reach, particularly during the rainy season when malaria incidence is highest. In rural Myanmar, it can take more than a day to reach malaria affected villages. Second, the diagnostic tests currently used in reactive case detection miss most *Plasmodium* infections because parasite densities are below the level of detection by rapid diagnostic tests [20]. These are critical limitations which have a major impact on the yield of programmes making use of reactive case detection [20–32]. A recent review of the published literature found that the yield from reactive case detection in the GMS, ranged from 0.1 to 4.2%, with predictably higher rates from the more sensitive PCR testing compared with microscopy and/or rapid diagnostic tests [33]. Overall approximately 200 contacts had to be tested by RDTs or microscopy to detect one *Plasmodium*-infected case [33]. These studies did not use uPCR and so would have missed a large proportion of asymptomatic infections. Even uPCR, with a limit of quantitation around 22 parasites/mL, does not detect all asymptomatic parasite carriers. It has been estimated that in the GMS uPCR would identify > 70% of the *P. falciparum* infections and > 85% of those infected with *P. vivax* [19]. These low parasitaemias are below the levels which generate transmissible densities of gametocytes at the time, but long-term follow up of malaria therapy patients and volunteers in malaria endemic countries indicate that these asymptomatic parasite densities oscillate and intermittently do produce transmissible densities [34–37].

The simplest and least expensive form of reactive case detection uses an RDT to screen contacts—which allows immediate treatment (i.e. a single encounter). This would have detected only 9 additional *Plasmodium* infected individuals following 1000 tested: 6 *P. falciparum* and 4 additional *P. vivax* cases among 1000 index cases. The substantially more labour-intensive conventional blood spot PCR which requires two visits to the village per case in reactive case detection (hoping that the asymptomatic infected individual identified by the PCR is contactable at or near home). This would have detected only 19 additional cases for 1000 index cases. Overall reactive case detection would have identified 5 (0.5%) of 1052 infected and hypothetically tested individuals. This comprised 0.4% of all 1223 identified infections. If PCR was used in reactive case detection this would have risen to 0.8% (10/1223). As uPCR identifies only 70–85% of all infected individuals, the true proportions of all infections identified are even lower. Thus, the likely impact of reactive case detection on the burden of malaria in these communities would have been negligible.

The extremely low yield of reactive case detection in this setting is contributed to by the ecology of malaria vectors in the GMS. Although there is some residual household transmission in the GMS evidenced by *Plasmodium* infections in young children who likely became infected in the household [15], the majority of *Plasmodium* infections in adults are contracted during work in or near forests [38]. Predominantly male groups camp for several days and nights in what remains of remote forests for a range of activities, the most lucrative of which is logging. Other activities include mixed groups of men and occasionally women harvesting forest flora and fauna. Following these activities, they return to their homes where there are few competent vectors and consequently little transmission.

In the GMS *P. vivax* is now the predominant human malaria species [39]. Treating *P. vivax* infections effectively requires schizontocidal treatment and a 7-to-14-day radical cure regimen with the 8-aminoquinoline primaquine to prevent relapse. Because 8-amioquinolines cause acute haemolysis in G6PD deficient individuals, G6PD testing is required before giving treatment. This is seldom available, although rapid tests to estimate enzyme activity have been developed and are being rolled out. The safety and adherence concerns associated with the radical cure of asymptomatic *P. vivax* infections are a further challenge to the utility of reactive case detection.

This study applied reactive case detection to the household of the index case, but there are alternative approaches to contact tracing. It is possible that more infections accumulate in households around the index household. Parker and co-workers explored the spatial distribution of *Plasmodium* infections in three of the villages in Western Cambodia preceding the MDAs described the current study [40]. Using the same uPCR survey data the authors linked cases with their geographical coordinates. Reactive case detection including neighbouring households around the household of the index case would only have detected a small proportion of cases unless the entire village was screened. An earlier exploration of the data collected in four villages along the Thai-Myanmar border included in the current study found that reactive case detection did not perform better than random screening and would only have detected additional cases by using a wide screening radius around the household of each index case, which basically includes the entire village [41].

The study is based on a unique dataset, which allows the detailed analysis of clustering of asymptomatic and symptomatic *Plasmodium* infections, but it has limitations. The prospective trial from which the data are derived was not designed to explore clustering. The analytic approach employed could have biased or cofounded the findings in unforeseen ways. Second, even uPCR misses 20 to 30% of asymptomatic infections, so the very small potential benefits of reactive case detection in reducing malaria are overestimated [19]. Third, the diagnostic sensitivity of the RDTs may have been overestimated, particularly for *P. vivax* infections, which would also have inflated the potential benefit of reactive case detection. Fourth, the study assumed all patent parasitaemias would cause symptoms and therefore provoke reactive case detection, but some may not have—further inflating its impact. Fifth, these villages were selected because they had relatively high prevalences of asymptomatic malaria parasite carriage. Thus, the probability of identifying cases by reactive detection would have been higher than in most other villages, so the overall meagre yield predicted from reactive case detection was likely higher than in most other villages. Sixth, although the study was large, and was conducted across the GMS region, it is possible that it was otherwise unrepresentative—although this does seem unlikely. Finally, this study only assessed households and did not screen neighbours, co-travellers, and co-workers which might have increased yields.

## Conclusion

Malaria elimination, by now the priority of national malaria control programmes in GMS, depends on the successful treatment of all or nearly all infections to interrupt transmission permanently. If the villages studied here are representative of the region then reactive case detection and its embodiment in the 1-3-7/CIFIR strategy will have a negligible impact on the malaria burden, but it has substantial cost in both human and financial resources. It may not even be feasible. Accessing many of the endemic villages during the rainy (malaria) season is a major operational challenge. Another difficult challenge, relapsing *P. vivax* (now the main cause of malaria) will require much greater use of radical cure, and it will not be solved by reactive case detection. With limited resources now, and potentially less international support in the near future, it is essential that available resources are used efficiently and effectively. Reactive case detection would likely be a waste of these precious resources. The key to malaria control and elimination in the region is the well-supported village health worker, but in foci of higher transmission in which a substantial proportion of the community has asymptomatic low density parasitaemia, mass drug administration is an effective elimination accelerator.

## Supplementary Information

**Additional file 1: Table S1.** Households with and without registered household numbers (Survey at M0).

## Data Availability

The data are available upon request to the Mahidol Oxford Tropical Medicine Research Unit Data Access Committee (http://www.tropmedres.ac/data-sharing) for researchers and following the Mahidol Oxford Tropical Medicine Research Unit data access policy (http://www.tropmedres.ac/_asset/file/datasharing-policy-v1-1.pdf). Queries and applications for datasets should be directed to Rita Chanviriyavuth (rita@tropmedes.ac). For the purpose of Open Access, the author has applied a CC BY public copyright licence to any Author Accepted Manuscript version arising from this submission.

## Abbreviations

CIFIR: Case investigation focus investigation and response
DP: Dihydroartemisinin-piperaquine (DHA-PPQ)
GMS: Greater Mekong Subregion
M[number]: Month [number]
MDA: Mass drug administration
RDT: Rapid diagnostic test; single low dose primaquine (SLD PQ)
TME: Targeted malaria elimination
uPCR: Ultrasensitive quantitative PCR
