# Supplementary material for: Clustering of malaria in households in the Greater Mekong Subregion: operational implications for reactive case detection
Source: Malar J. 2021 Aug 26;20:351. doi: 10.1186/s12936-021-03879-9 (PMC8393740; doi:10.1186/s12936-021-03879-9)
Supplement: Supplementary file 1 — Additional file 1: Table S1. Households with and without registered household numbers (Survey at M0). [file 12936_2021_3879_MOESM1_ESM.docx]

**Appendix:**

Table S1: Households with and without registered household numbers (Survey at M0)

**Table S1: Infected individuals (with uPCR confirmed result) in households with and without registered household numbers (**Survey at M0)

A: Those with household numbers

|  | Pf only | Pv only | Pf + Pv | P spp. | Total |
| --- | --- | --- | --- | --- | --- |
| All *Plasmodium* infections | 289 | 557 | 125 | 252 | 1,223 |
| Patent | 88 | 50 | 25 | 8 | 171 |
| Sub-patent | 201 | 507 | 100 | 244 | 1,052 |
| Asymptomatic with density  (>22/mL) reported) | 190 | 474 | 98 | 201 | 963 |
| Density >50,000/mL | 67 | 89 | 56 | 17 | 229 |
| Density >5,000/mL | 112 | 222 | 75 | 35 | 444 |
| Density ≤5,000/mL | 78 | 252 | 23 | 166 | 519 |

Pf = *P. falciparum*, Pv = *P. vivax*, *P. spp* = *Plasmodium* species - there was insufficient DNA for species identification or no amplification was obtained

B: Those without a household number

|  | Pf only | Pv only | Pf + Pv | P spp. | Total |
| --- | --- | --- | --- | --- | --- |
| All *Plasmodium* infections | 2 | 10 | 1 | 6 | 19 |
| Patent | 0 | 0 | 0 | 0 | 0 |
| Sub-patent | 2 | 10 | 1 | 6 | 19 |
| Asymptomatic with density  (>22/mL) reported) | 2 | 8 | 0 | 6 | 16 |
| Density >50,000/mL | 1 | 3 | 0 | 0 | 4 |
| Density >5,000/mL | 1 | 3 | 0 | 0 | 4 |
| Density ≤5,000/mL | 1 | 5 | 0 | 6 | 12 |

Pf = *P. falciparum*, Pv = *P. vivax*, *P. spp* = *Plasmodium* species - there was insufficient DNA for species identification or no amplification was obtained
